# Ethical issues in managing Lysosomal storage disorders in children in low and middle income countries

**DOI:** 10.12669/pjms.334.12975

**Published:** 2017

**Authors:** Bushra Afroze, Nick Brown

**Affiliations:** 1 Dr. BushraAfroze, FCPS, Department of Paediatrics& Child Health, The Aga Khan University Hospital, Karachi, Pakistan; 2 Dr. Nick Brown, MCPCH, Department of Paediatrics, Salisbury District Hospital, Salisbury, UK

**Keywords:** Antenatal screening, Cascade screening, Ethics, treatment, Low and middle income countries, Lysosomal storage disorders

## Abstract

The lysosomal storage diseases are a group of rare, inherited metabolic diseases affecting about 1 in 7000 to 8000 people. In recent years, the introduction of enzyme replacement therapy, substrate reduction therapy and small molecule therapy, has changed the natural course of this otherwise progressive group of disorders leading to severe morbidity and early mortality. These treatment options, however, are extremely expensive and are needed for life thus presenting an economical as well as ethical challenge to the affected families and the health care system of a country. This paper presents a case for the prevention of the lysosomal storage disorders as a model for other inherited metabolic disorders in the form of antenatal testing and cascade screeningfor couples and families at risk of having affected off-springs and compares it to the cost incurred on the enzyme replacement therapy in the backdrop of the health care prioritiesof Pakistan, a low middle income country. Similar economic and ethical challenges are faced by most low and middle income countries. The literature search was done using Pubmed and Clinical trials databases using key words: “Lysosomal storage disorders”, “natural course”, “ethics”, “cascade screening”, “Thalassemia” and “cascade screening”. A total of 225 articles in English language were scanned from 1980-2016, 80 articles describing the natural course of LSD with and without ERT, ethical issues related to the treatment of LSD and strategies employed for the prevention of genetic disorders were prioritized.

## INTRODUCTION

Lysosomal storage disorders (LSDs) are a group of heterogeneous inherited metabolic disorders, comprising of 50 distinctivedisorders.^1^ Various mechanisms including; defects in lysosomal exocytosis, reduced lysosomal catabolic efficacy, defects in lysosomal transport machinery, impairment in lysosomal metabolite efflux or dysfunction of lysosomal integral membrane proteins can result in multisystemic involvement. The natural course of most LSDs is a progressive one resulting in significant morbidity and reduced life expectancy. The severity of the disorders is also variable with symptomsranging from in-utero hydropsfetalis tosubtle adult symptoms. The overall prevalence of LSDs has been estimated as 1 in 7000 to 1 in 8000 live births.^2^,^3^ However, considerable variations are reported in various surveys conducted in different countries^4^,^5^ and data on the prevalence of LSDs inmost low and middle income countries (LMICs) like Pakistan is scarce. Two hospital based studies by Afroze *et al* and Cheema *et al*. have reported 21 and 59 LSDs respectively. Afroze *et al* screened 426 patients for inherited metabolic disorders (IMDs), out of which 85 patients were confirmed to have various IMDs. Among the confirmed IMDs patients, 21 patients were reported with LSD.^6^ Cheema *et al*. screened239 patients for IMDs and 180 patients were confirmed to have various IMDs. Among the confirmed IMDs patients, 59 patients with LSD^7^ were reported.

The World Bank classifies 135 countries as LMICs among 215 countries based on the national gross income per capita.^8^ It is estimated that one billion of the current global population live in communities with a preference for consanguineous marriage.^9^ Autosomal recessive diseases are more common in communities with the tradition of consanguineous marriages. All LSDs are inherited as autosomal recessive (AR) disorders; except for Mucoploysaccaridosis Type-II, Fabry disease and Danon disease, which are inherited as X-linked recessive disorders.

Like all other AR disorders, the prevalence of LSDs is expected to be high in LMICs like Pakistan due to high consanguinity rate of 62.7%.^10^ Seven LSDs have Food and Drug Administration (FDA) approved treatment and active research is underway for few others. However, these treatment options are very expensive and there is an exponential increase in cost, as the number to treat increases every year on year with improved survival. The doses needed to treat are weight based, therefore; inevitably increase as the children grow into adults. Therefore; rational treatment for LSDs is challenging even in High Income Countries (HIC), let alone in LMICs. The focus of this paper is to present the existing oremerging therapeutic options for various LSDs and compare the cost effectiveness of life-long treatment for treatable LSDs to the preventive reproductive options for high risk families to have children with LSDs in the back drop of economic conditions and health care priorities of a low income country like Pakistan. Similar ethical challenges are faced by most LMICs in allocating health care resources to treat rare diseases like LSDs. Countries like Pakistan and other LMICs have to decide whether they can even begin treatment for rare disorders like LSDs at all, whether a treatment programme might be possible if it is introduced alongside a programme of family cascade screening, to make recurrenceless likely in the immediate and the extended family.

### Theraputic options for lysosomal storage disorders; past, present and future

Gaucher disease was the first LSD to be described by Philippe Gauche in 1882^11^, followed by Fabry disease in 1898, independently described by Johannes Anderson^12^ and Fabry.^13^ Gaucher disease was the first LSD, for which the recombinant DNA-produced analogue of human β-glucocerebrosidase enzyme was developed and was approved by the FDA in 1991. Since then, impressive research has been undertaken to develop various therapeutic options for other LSDs. These include; enzyme replacement therapy (ERT), substrate reduction therapy (SRT), small molecules treatment to facilitating intracellular substrate transport, chemical chaperones, gene therapy, stem cell therapy, and others including adjunctive therapies.^14^

ERT has been the most successful technology to date as a treatment for LSDs followed by small molecule therapy and SRT. The basic principal of ERT involves the intravenous administration of fully functional enzyme that is taken up by the cells and then delivered to the lysosomes, where the delivered enzyme compensates for the deficient enzyme. InSRT, small molecule drugs are ingested orally, which inhibit the first step in glycosphingolipid biosynthesis, aiming to reduce the rate of glycosphingolipid biosynthesis resulting in reduction of the catabolic effects. Small molecule therapy either facilitates intracellular substrate transport or prohibits pathways triggering apoptosis.

Between1983 and 2013, fourteen drugs for seven LSDs received FDA approval. These include; ten ERT, three small molecule therapy and one SRT (14). For Gaucher disease; four ERT and one SRT have been approved, whereas for Pompe disease; two ERT and for Cystinosis; three SRT are included in the FDA approved drugs for LSDs.

Active research to develop effective treatment for more LSDs is underway. The LSDs for which clinical trials are in various phases include Mucopolysaccharidosis Type-IIIA, MucoploysaccharidosisType-IVA, Mucoploysaccharidosis Type-VII, Metachromatic Leukodystrophy, Wolman Disease, Alpha-mannosidosis and Niemann Pick C/D. The drugs being investigated in clinical trials are summarized in Table-I.

**Table-I:** LSDs treatment options under clinical trials.

*Disease*	*Drug*	*Category*	*Clinical Trial Phase*
Mucoploysaccharidosis-IIIA	Recombinant human Heparan-N-Sulfatase	ERT	Phase 2
Mucopolysaccharidosis-IVA	Recombinant human N-acetylgalactosamine-6-sulfatase (BMN 110)	ERT	Phase 3
Mucopolysaccharidosis-VII	Recombinant human β-glucuronidase (UX003)	ERT	Phase 3
Metachtomatic Leukodystrophy	Recombinant human Arylsulfastase A	ERT	Phase 1/2
Wolman Disease	Sebelipase (SBC-102)	ERT	Phase 3
Alpha-mannosidosis	Lamazyme	ERT	Phase 2
Niemann Pick C/D	N-Acetylcysteine	SRT	Phase 1/2
	Migulstat	SRT	Phase 2
	2-hydroxy-proyl-beta-cyclodextrin	SRT	Phase 2

### Cost of enzyme replacement therapy for lysosomal storage disorders in the back drop of Pakistan economic and health care priorities

Over the last quarter of the century, the evolution of treatment of LSDs has been enormous. However, the health service cost incurred by ERT is daunting even in HICs. AsBeutlercommented in apaper written shortly after Cerezyme^®^ received FDA approval, as ERT for Gaucher Disease “The high cost of alglucerase has created difficult problems for patients in developed countries and impossible ones in under-developed countries”.^15^ Children in the UK are relatively fortunate in that treatment for IMD (neurometabolic disorders, mitochondrial disorders and congenital disorders of glycosylation) is funded centrally as a ‘Highly Specialized Services’ (AGNSS).^16^

After ERT, the natural course of the LSD is ameliorated, the disease progression is halted thus the complications of untreated disease causing death are modified leading to a near normal life expectancy. Thus, the ERT becomes a life-long requirement with an exponential increase in the cost of treatment, which increase every year as the children grow into adults. The estimated annual cost of some of the FDA approved ERT for treatable LSDs for a patient of 10 Kg are shown in Table-II. The annual ERT cost for a 10 kg patient with MPS-I is USD 67,600/year, at 40Kg it is USD 270,400/year and at 60 Kg, itis USD 405,600/year. Other costs include;portacath insertion for weekly or fortnightly ERT infusions, cost of consumables used for the intravenous infusions; day-care hospitalization cost for each infusion and travel cost to hospitals. There are potential infusion related adverse events(pyrexia, chills, hypertension, tachycardia, cutaneous reactions, headache, nausea, fatigue, malaise, joint pain, dyspnea, facial edema, dizziness and bronchospasm) for all seven FDA approved ERT products and the black box warnings (life-threatening anaphylactic reactions) for four FDA approved ERT products alglucosidasealfa - Myozyme^®^ and Lumizyme^®^, idursulfase-Elaprase^®^, laronidase -Aldurazyme^®^).^17^ Early infusions must be given by physiciansexperienced in dealing the ERT. The ERT for LSDs is either weekly or two weekly. Therefore; in developed countries like the UKand European countries^18^ with home health care facilities, after successful ERT initiation in hospitals, later infusions are continued at home. This shift of hospital infusions to home infusions for ERT is even more challenging in most LMICs like Pakistan due to lack of home health care facilities capable of dealing with potential life-threatening adverse effects of the ERT. Thus, a patient will be admitted weekly or two weekly depending on the underlying LSD for which ERT has been commenced. Not only this, most LSDs require lifelong multi-disciplinary teamcare. A patient with MPS-I will need an ENT specialist, a cardiologist, a dentist, a general surgeon, anorthopedic surgeon, a neuro-surgeon, an ophthalmologist, a developmental specialist, a pulmonologist, a psychologist, a geneticist and an ERT team of nurses.

**Table-II:** Estimated annual treatment cost for some FDA approved ERT for LSDs.

*Disease*	*Product*	*Dose per kg body weight*	*Annual cost for 10 kg*
Mucoploysaccharidosis-I	Aldurazyme^®^	0.58 mg	USD 67,600/-
Mucopolysaccharidosis-II	Elaprase^®^	0.5mg	USD 173,182/-
Mucopolysaccharidosis-VI	Naglazyme^®^	1 mg	USD 214,906/-
Gaucher Disease	Cerezyme^®^	60 IU	USD 64,350/-
Pompe Disease	Myozyme^®^	20 mg	USD 78,000/-
Fabry Disease	Fabrazyme^®^	1 mg	USD 40,000/-

In the ERT era, many physicians consider ERT as essential for all patients with Gaucher disease. Studies on the natural of Gaucher disease has shown little or no progression in patients with the homozygous c.1226A->G (N370S) mutation, variable progression in patients compound heterozygotes for a mild mutation such as N370S and a more severe mutation such as c.1448G->C (L444P), onset of symptoms at much later age in patients with homozygous N370S mutation.^19^-^24^

These issues must be seen in the economic and ethical context of more prevalent diseases with greater population burden of disease. In over half of LMICs, at least 75 children out of every 1000 live births died before reaching their fifth birthday.^25^ Pakistan’s mortality rate for children under five year is 81/1000 live births.^26^ The leading causes of under-fivemortality being; birth asphyxia (22 %), sepsis (14 %), pneumonia (13 %), diarrhoea (11 %) and prematurity (9 %).^27^ Annual cost incurred on treating a single patient with MPS-I of 10 Kg weight is equivalent to treating an estimated 8,894 patients with pneumonia in hospital setting.^28^ As health care budgets are inelastic money spent in one segment of the health care economy must be taken from another segment. Thus, health setting priorities in LMICs like Pakistan demand justified allocation of the already scanty funds allocated to the field of health. Setting health care priorities may be considered political unpopular but has been practiced in the USA including Georgia, Hawaii, Minnesota and Oregon.^29^

### Ethics of and options for prevention of lysosomal storage disorders through antenatal diagnosis and cascade screening

Most LSDs are inherited in an AR manner and, therefore, have a 25% risk of recurrence in each pregnancy for couples with a previously diagnosed child. Molecular characterization of the particular LSD in a family, allows the couples to choose an appropriate reproductive option most suitable for them including prenatal genetic testing followed by selective termination of the affected fetus or intro-vitro fertilization with pre-implantation genetic diagnosis. Legal status for selective termination of pregnancy (TOP) varies among countries and may not be clearly defined in some LMICs. The law in Pakistan allows TOP under section 338 of the Pakistan Penal code, which was revised by the government of Pakistan in 1990. Under this clause, the conditions for legal abortion depend on the developmental stage of the fetus; whether the fetus’s organs are formed or not. Islamic scholars have usually considered the fetus’s organs to be formed by 120th day of gestation. Before 120 days of gestation, abortions are permitted to save the woman’s life or in order to provide “necessary treatment.” However, the term “necessary treatment” is not clearly defined in law and is open for interpretation.^30^

The estimated cost of antenatal testing for LSDs in Pakistan is about USD 2,500 -3,000, which spares a family from the emotional, social and life-long financial pressure to look after a child with LSD in a setting, where health is paid completely out of pocket. This cost is though beyond the reach for many families but is a one-time expense and much lessthan the potential life-long expense. If prevention of LSDs in the form of antenatal diagnosis followed by selective TOP in case of affected fetus is not practised, a family can have a secondaffected child with the ensuing implications for morbidity and financial burden. Contrary to the common belief, theoption of selective TOP is already practised for genetic disorders in Pakistan. A 22% voluntary TOP, based on parents’ anxiety and fear of having another child with agenetic disorder even in the absence of an antenatal diagnosis has been reported.^31^

Further prevention of thenew patients born with a life-long expensive disease like LSD can be prevented by “Cascade Screening”. Cascade screening involves carrier testing for the siblings of the progeny and parents’ siblings for a genetic disease prevailing in that particular family. This is a proven method adopted to evaluate carrier status for individuals in at-risk population. This allows offering effective pre-marital genetic counseling, through which carriers make “informed decisions” to choose their potential spouses. Thus relatives of each patient get an opportunity to prevent the birth of other children with that disease. This method is a recognized way ofdecreasing disease burden of AR diseases in societies that practice endogamy. A similar approach has been applied in Pakistan for the prevention of beta-thalassemia, a common AR genetic disorder there.^32^ Though it raises ethical issues of “negative eugenics”, but is a pragmatic solution for unaffordable expensive genetic disorders like LSDs in the backdrop of the health care priorities in LMICs. In 1998, World Health Organization (WHO) published guidelines for genetic testing, according to which no compulsory genetic testing should be carried out.^33^ However countries including Iran, Saudi Arabia, Palestine and Cyprus have laws in place making premarital screening for haemoglobinopathies mandatory for all couples before they are given approval to get married.^34^-^38^

The WHO has recommended the implementation of community genetics programs in LMICs. The focus of these recommendations is the prevention of congenital disorders and genetic diseases at the population level, in addition to providing genetics services, including diagnosis and counseling, for individuals and families. At present, these include newborn screening for congenital hypothyroidism and phenylketonuria where immediate intervention can prevent neurocognitive harm and population screening for carrier detection for common recessive conditions, such as sickle cell anemia and alpha-thalassemia.^39^ There is immense potential to broaden the horizon of population screening for specific AR diseases, which are more prevalent in the specific communities, but, economically possible only if the disease prevalence for common genetic disorders is known in that population. The newborn screening for genetic disorders serves an important tool to provide this information.

Structure of carrier screening programmes varies among different populations in several aspects, including whether the programmes are mandatory or voluntary, at which stage of carrier screening the education and counseling was provided; pre-marital, pre-pregnancy or antenatal, and whether screening test is offered pre-marital, pre-pregnancy or antenatally. In 1973, the Cypriot government started pre-marital carrier screening and counseling for thalassemia, which was actively supported by the CypriotOrthodox Church. After this introduction, the number of affected births dropped from 51/1000 in 1974 to 8/1000 in 1979.^37^ By the 1990s, the rate was 5/1000 births, between 2002 and 2007, no affected births.^40^ The government of Pakistan passed the bill “Compulsory Blood Test of the Relatives of Thalassemia Patient Act 2014” in its’ National Assembly as an attempt to reduce the thalassemia burden in the country. The success of such measures in any country is affected by cultural, social and religious beliefs of the individuals and the society. The individuals’ ethical right of making “informed choices” cannot be denied by any law or any country.

In conclusion, IMDs are an example of the role in which paediatricians and obstetricians in LMICs can guidetheir governments and funding agencies in selecting the most altruistic health care priorities. Beutler commented in his paper “everyone wants to see the benefits of any possible treatment extended to the ill, the delivery of health care is unfortunately a “zero-sum game.” Means must be found to provide treatment, within reason, to patients with rare genetic diseases without depriving large numbers of deserving individuals of basic health care needs. The first step is recognition of the problem”.^41^

### Author’s Contribution

**BA** conceived, designed, did literature search and wrote the manuscript.

**NB** did review and intellectual input in the manuscript.
